# Prevalence and Trends of Obesity and Association with Socioeconomic Status in Thai Adults: National Health Examination Surveys, 1991–2009

**DOI:** 10.1155/2014/410259

**Published:** 2014-03-17

**Authors:** Wichai Aekplakorn, Rungkarn Inthawong, Pattapong Kessomboon, Rassamee Sangthong, Suwat Chariyalertsak, Panwadee Putwatana, Surasak Taneepanichskul

**Affiliations:** ^1^Department of Community Medicine, Faculty of Medicine, Ramathibodi Hospital, Mahidol University, Rama VI Road, Rajdhevi, Bangkok 10400, Thailand; ^2^National Health Examination Survey Office, Nonthaburi 11000, Thailand; ^3^Faculty of Medicine, Khon Kaen University, Khon Kaen 40002, Thailand; ^4^Epidemiology Unit, Faculty of Medicine, Prince of Songkla University, Songkhla 90110, Thailand; ^5^Faculty of Medicine, Chiang Mai University, Chiang Mai 50002, Thailand; ^6^Ramathibodi School of Nursing, Faculty of Medicine, Ramathibodi Hospital, Mahidol University, Bangkok 10400, Thailand; ^7^College of Public Health Sciences, Chulalongkorn University, Bangkok 10330, Thailand

## Abstract

We determined the prevalence of obesity in Thai adults aged 20 and over in 2009 and examined trends of body mass index (BMI) between 1991 and 2009. Data from Thai National Health Examination Survey for 19,181 adults in 2009 and 64,480 adults between 1991 and 2004 were used to calculate age-adjusted mean and prevalence. Logistic regression was used to examine the association of obesity with education level. In 2009, age-adjusted prevalence of obesity classes I (BMI 25–29.9 kg/m^2^) and II (BMI ≥30 kg/m^2^) in Thai adults aged ≥20 years were 26.0% and 9.0%, respectively. Compared with primary education, the odds of obesity class I were highest in men with university education. For women, the odds of obesity classes I and II were highest in those with primary education. BMI significantly increased from 21.6 kg/m^2^ in men and 22.8 kg/m^2^ in women in 1991 to 23.3 kg/m^2^ and 24.4 kg/m^2^ in 2009, respectively. The average BMI increases per decade were highest in men with secondary education (1.0 kg/m^2^, *P* < 0.001) and in women with primary education with the same rate. There were increasing trends in BMI with slight variation by SES groups in Thai men and women during 1991–2009.

## 1. Introduction

The prevalence of obesity has been increasing worldwide. The estimated number of population with obesity around the world is 1.5 billion in 2012 and it continues to rise [1]. The increases of obesity affect all classes of socioeconomic status (SES) with certain difference in both developed countries and developing countries in recent decades [1, 2]. As a result, burdens of diseases from chronic noncommunicable diseases associated with obesity such as cardiovascular diseases, diabetes, metabolic syndrome, and hypertension are increasing [3, 4]. The World Health Organization has recently set obesity as one of the key indicators for global action on noncommunicable diseases [5]. Studies have shown that the direction of association between obesity and SES varied by population and economic status of the countries. In the developed countries, individuals with lower socioeconomic status were more likely to be obese than those in the higher socioeconomic group [6, 7]. For the developing world where countries are in the transition of epidemiological period, pattern of obesity has varied by gender and socioeconomic status. The reversal association between SES and obesity in developing countries has been observed earlier in women [7].

In Thailand, a previous national health examination study showed that the prevalence of obesity increased approximately by 60% during 1991–2004 [8]. The increased prevalence was higher in urban than in rural areas in both sexes. The surveys also reported that the distribution of overweight and obesity varied by education level, with significantly higher prevalence in men with high education level, but in women with low education level. It is not clear whether the pattern has been changed in the recent national health survey. In the present study, we reported the prevalence of the latest national health examination survey in 2009 and the trends of body mass index and prevalence of obesity from 1991 to 2009. We also examined the pattern of association between obesity and education level by sex, age group, and area of residence during 1991–2009.

## 2. Methods

NHES is nationally representative of health examination survey of Thai population conducted in 1990, 1997, 2004, and 2009. The survey was conducted by the National Health Examination Survey Office, Health System Research Institute, Thailand. In each survey, a multistage cluster sampling was employed. The sampling technique has been described elsewhere [9, 10]. For the NHES IV, briefly, the sampling unit in the first stage was province in each region, the second was the district, and the third stage was village in rural areas and enumeration unit in urban areas. The final stage was individuals in sex and age-specific group. The sample size was targeted at 21,960 participants aged ≥15 years. The final sample size collected was 20,450 with a response rate of 93.1%. In this study, we included those aged 20 years and over with a total of 19,181 in the analysis. The sample sizes for the 1990, 1997, and 2004 surveys were, 15124, 7726, and 41630, respectively. This study was approved by the Ethical Review Committee for Research in Human Subjects, Faculty of Medicine, Ramathibodi Hospital, Mahidol University.

### 2.1. Data Collection and Anthropometric Measurement

Data on weight and height were measured using standard procedures [11]. Weight was measured while participants wore light cloths; height was measured at standing without shoes. Body mass index was calculated as weight in kilograms divided by height in meters squared.

Obesity was defined using criteria for Asian population, at a cut-off point of BMI ≥25 kg/m^2^ as obesity due to the higher risk of developing diabetes and obesity-related diseases compared to western population [12]. Consequently, BMI was divided into 4 categories: overweight: BMI 23–<25, obesity class I: BMI 25–<30, and obesity class II: BMI ≥30 kg/m^2^. Education was categorized into four groups: less than primary, primary, secondary or vocational, and university education. Self-reported smoking status was categorized as current smoker and nonsmoker.

### 2.2. Statistical Methods

All the statistical analyses were taken into account the complex survey design using STATA software 10.1 (stat Corp. Texas). In the analysis of the year 2009, age-adjusted mean of BMI and age-adjusted prevalence of obesity were calculated according to sex, area of residence (urban/rural), and level of education. The age and sex adjusted mean and prevalence of obesity were standardized using the standard population of the estimated 2004 population. For the 2009 survey, multinomial logistic regression models were used to assess the association of the ordinal scale of BMI categories: overweight and obesity class I and obesity class II with independent variables of educational levels controlling for age, smoking, area of residence, and geographic regions (north, northeastern, central, south, and Bangkok). We assessed the interaction by adding multiplicative interaction terms of area of residence and indicators variables for education levels in the models and found no significant interaction at *P* value <0.10. In the trend analysis between 1991 and 2009, we restricted the age group to those 20–59 years old, because the BMI data for those aged ≥60 years were not available in the 1997 survey. Sample size for those aged 20–59 years in each survey included a total of 11,218, 3,062, 19,962, and 10,103 for years 1991, 1997, 2004, and 2009, respectively. We used linear regression to evaluate the average change of BMI per decade by using BMI of each survey as dependent variable and the survey year as independent variable controlling for age, area of residence, and educational levels. Logistic regression was used to examine the linear trends in sex-specific prevalence of overweight and obesity class I and class II, separately over the four surveys by educational level with year of survey as a continuous variable controlling for age and area of residence. All the models were run separately for men and women. The odds ratios for 1-year change in the prevalence were reported. Statistical significance tests between groups and years were compared using the adjusted Wald test. Statistical significance was considered at 2 sides with *P* value <0.5.

## 3. Results

### 3.1. BMI and Prevalence of Overweight and Obesity in 2009

In 2009, overall, age-adjusted mean BMI among Thai adults aged ≥20 years was 23.9 kg/m^2^ (95% CI 23.6, 24.2 kg/m^2^). Women had higher BMI than men (24.4 kg/m^2^ (95% CI 24.1, 24.8) versus 23.3 kg/m^2^ (95% CI 23.0, 23.6), *P* < 0.001). Age-adjusted prevalence of overweight, obesity class I, and obesity class II was 17.5% (95% CI 16.7, 18.4%), 26.0% (95% CI 24.1, 28.0%), and 9.0% (95% CI 7.9, 10.2%), respectively. The corresponding prevalence, except for overweight, was higher in women than in men (17.0% (95% CI 16.1, 17.9%), 29.0% (95% CI 26.5, 31.6%), and 11.5% (95% CI 10.1, 12.9%) in women and 18.2% (95 CI 16.8, 19.6%), 22.8% (95% CI 20.1, 25.7%), and 6.3% (95% CI 5.1, 7.6%) in men, resp., all *P* values <0.05).  Table 1 shows the prevalence of overweight and obesity overall and by sex, age, area of residence, and educational levels. For men, obesity class I and class II prevalence were significantly higher in urban than in rural areas (all *P* < 0.001); however, for women, only obesity class II prevalence was significantly higher in urban than in rural areas (*P* = 0.006). The pattern of obesity prevalence by education levels varied according to sex. Among men in rural areas, the prevalence of obesity class I was higher among those with higher education levels and was highest among the university group, but, among men in urban areas, the prevalence of the obesity class I was relatively uniform by educational levels. The obesity class II prevalence in men was not significantly different across educational levels. For women, there was no significant difference in prevalence of obesity class I and class II across educational levels; however, the prevalence of obesity class I was highest in the primary education level.  Table 2 shows adjusted odds ratios of overweight and obesity associated with age, areas of residence, and educational levels in 2009. After controlling for age and area of residence, for men, education was positively associated with overweight and obesity class I with highest odds ratios among those with university education but was not significantly associated with obesity class II. For women, the adjusted odds of overweight and obesity appeared to be significantly highest in the primary education group and lowest in the university education group as compared to the less than primary education group.

### 3.2. Trends in Overweight and Obesity

**Figure 1:**
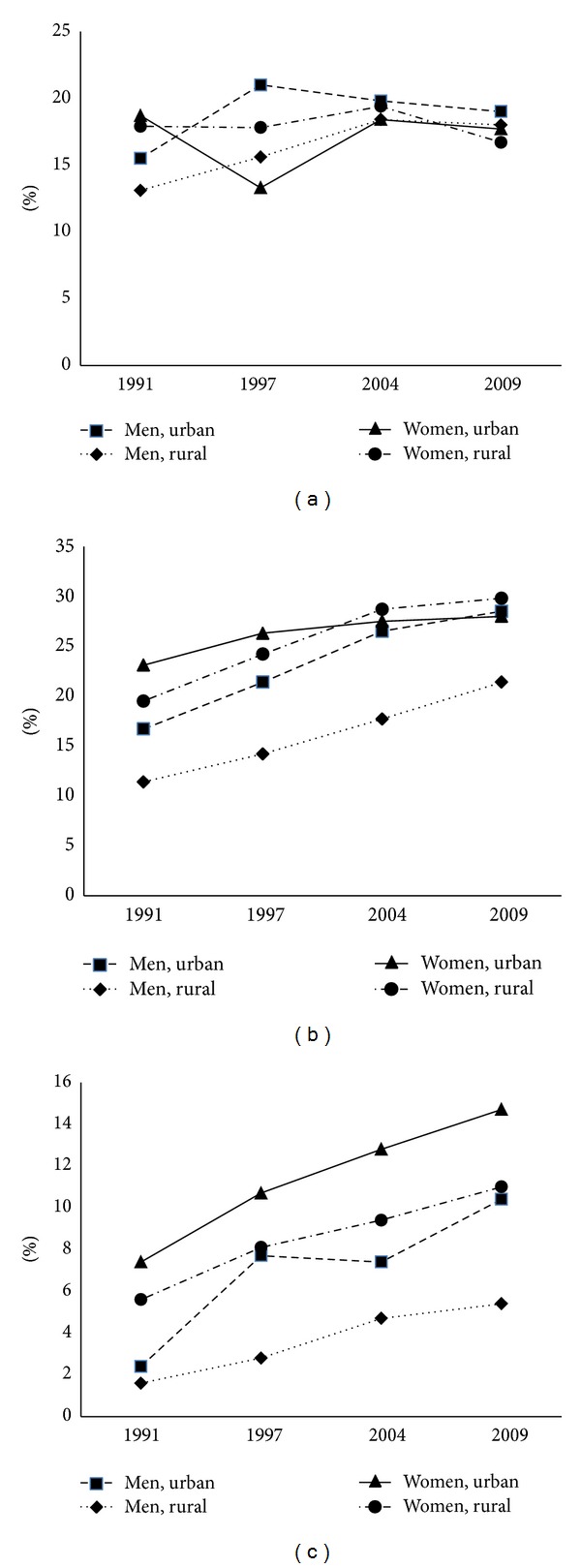
Age-adjusted prevalence of BMI categories: (a) BMI 23–<25 kg/m^2^, (b) BMI 25–<30 kg/m^2^, and (c) BMI ≥30 kg/m^2^ by sex and area of residence among Thai adults aged ≥20–59 years, Thai NHES 1991–2009.

During 1991 and 2009, the overall age-adjusted prevalence of obesity class I and class II in Thai adults aged 20–59 years increased significantly by the year of survey, whereas overweight prevalence was relatively stable. For men, the prevalence of obesity class I increased from 12.5% in 1991 to 16.6% in 1997, 19.9% in 2004, and 23.5% in 2009, and the corresponding prevalence of obesity class II was 1.7%, 4.3%, 5.4%, and 6.8%, respectively. For women, the corresponding prevalence for obesity class I was 20.2%, 24.9%, 28.5%, and 29.4% and for obesity class II was 5.9%, 8.8%, 10.3%, and 12.1%, respectively.  Figure 1 shows the trends in prevalence for men and women in urban/rural areas. Obesity class I and class II prevalence for all subgroups, except for women in urban areas, increased significantly across 1991–2009.

**Figure 2:**
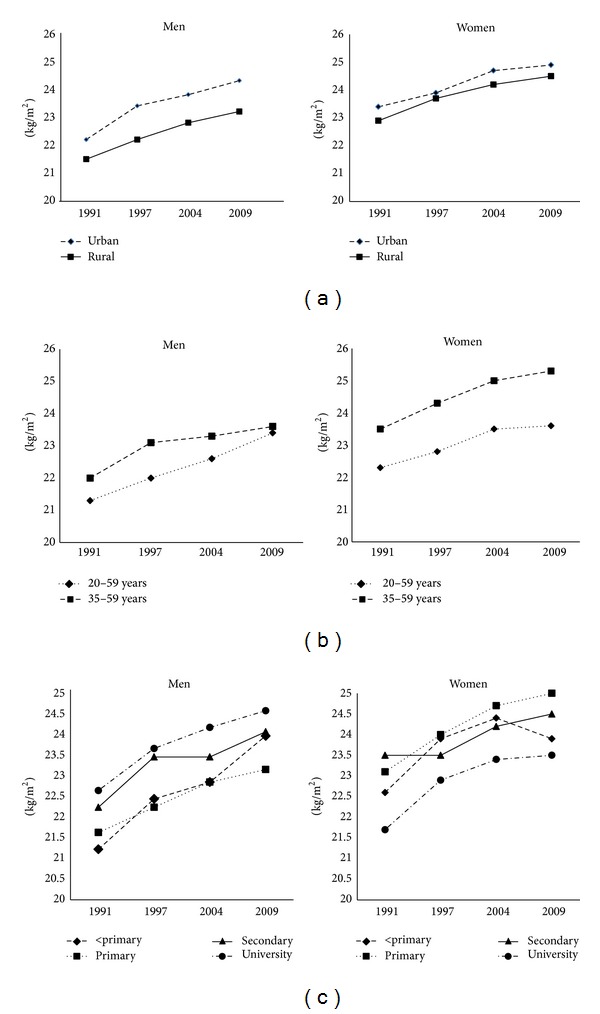
Age-adjusted mean BMI by sex, area of residence (a), age group (b), and education levels (c) in Thai adults aged ≥ 20–59 years, Thai NHES 1991–2009.

Figure 2 shows increasing trends in age-adjusted mean BMI by survey year according to sex, area of residence, age groups, and education levels. Overall, the BMI trends increased for all subgroups with certain extent. In men, the adjusted mean BMI increased from 21.6 kg/m^2^ in 1991 to 23.3 kg/m^2^ in 2009, and the corresponding mean BMI in women was 22.8 kg/m^2^ and 24.4 kg/m^2^, respectively. In linear regression analysis, the average increased BMI per decade was 0.8 kg/m^2^ (*P* < 0.001) for men and 0.9 kg/m^2^ (*P* < 0.001) for women. The mean BMI increased across all educational levels. For men, the rates of increase were highest among those with secondary education of 1.0 kg/m^2^ per decade and for women with primary education group with the same rate. Women with university attainment had the lowest rate of increase in BMI (0.7 kg/m^2^) per decade.

Table 3 shows that there were significant increases in annual prevalence odds of obesity class I and class II in both men and women between 1991 and 2009. According to educational levels, for men, increases of obesity class I were significant for those with primary and secondary education levels (*P* < 0.001 and 0.002, resp.) and increases in obesity class II were significant for both with less than primary education and secondary education group (*P* = 0.03 and 0.02, resp.). Among women, for obesity class I, the increase was significant in the primary education group (*P* < 0.001) and for obesity class II was significant in both primary and secondary education groups (<0.001 and 0.012, resp.).

## 4. Discussion

The prevalence of overweight and obesity defined by BMI in Thai population from 1991 to 2009 linearly increased with an average of 0.95 kg/m^2^ per decade and affected all SES groups. Compared to previous surveys, the prevalence as well as mean BMI increased dramatically during 1991–2009 with no sign of leveling off. The average increased BMI was higher than that of the global increase of 0.4-0.5 kg/m^2^ and was one of the highest among the Southeast Asian countries with an average increase per decade of 0.7 kg/m^2^ in men and 1.0 kg/m^2^ in women [1]. With regard to SES classes, in 2009, obesity class I (BMI 25–29.9 kg/m^2^) was positively associated with higher education in men but was negatively associated in women. However, the higher annual increment in mean BMI and obesity class I was found in the primary education level in both men and women. This might suggest that there is a tendency of a shift in obesity toward the lower educational group in men in the near future.

Compared to other countries in Asia, the rise in BMI and prevalence of obesity in Thailand was consistent with the findings of other Asian countries [13–15]. In low income and middle income countries, individuals in the high SES urban areas are the first to have high prevalence of obesity and the prevalence shifts to the lower SES as economic growth increases. The pattern of shift in women concurred with studies in other middle income countries where obesity rapidly increases in the lowest income groups [2, 12, 16, 17]. The lower obesity among men in low SES has been explained and shared by the common nature that men in the lower SES were in occupation with higher energy expenditure [6, 7, 18]. The more affluent men have greater access to food supply and are less physically active. In addition, the cultural preference of fat body shape among men also plays role, as a larger body size is more likely to be valued as a sign of prowess [6]. Education might be a protective factor for people in high income countries, and for women in low or middle countries, but it might hardly apply for men. Studies in several countries revealed that the most common association pattern was the nonsignificant or curvilinear relationship among men particularly in medium and high human index countries with a higher percentage of countries in medium Human Development Index having a positive relationship [18, 19]. The higher prevalence of obesity among the primary education women might also reflect inequity in knowledge and access to healthy lifestyle, as women in the lower education are less aware and accessible to better food choice. However, in developing countries, it is less clear whether there are differences in energy expenditure and a trend towards less physical activity and less concern to have leisure-time exercise. Research about the influence of lifestyle and obesogenic environment on obesity associated with SES in developing countries deserves further study.

Although there are limited studies to explain the casual factor of the current increasing trends in Thai population, imbalance of energy intake and expenditures, in general, are implicated in the rising of obesity [16]. The association of urbanization with higher obesity prevalence had been reported in our previous studies and others [13, 16]. Globalization of the fast food and processed food makes the cheap and high energy food more accessible throughout the country [16]. Availability of food due to reduction in the cost of food has been implicated as a major driver of increase of the global obesity during the past 2 decades, and Thailand is no exception [16]. The Thai Gross National Product (GNP) has continuously grown from million Thai baht 2,082 in 1991 to 3,008, 3,278, and 4064 in the years 1997, 2004, and 2009, respectively [20]. The lack of excess food consumption to the poor becomes uncommon, although lack of access to healthy food is possible. The poor choice might be due to access to information related to healthy food and less health concern or unawareness of the association between health consequences of excess energy intake. Thus, low SES groups can easily access cheap high energy diet which leads to gain weight. Increases in food supply are the major determinants of weight gain of the populations [21]. In some middle income countries, the concurrent trends in adoption of knowledge of obesity harm to health might weaken the positive relationship between SES and obesity; however, this is still not the case in men with higher education in Thai population [2, 6, 7].

There are limited data for interventions of obesity in low and middle income countries. Interventions to prevention and control obesity need system approach [22]. Multifaceted initiatives and multisectoral coordination across several sectors of government, NGO, industries, and civil society are needed. Currently, the Thai Ministry of Public Health has launched a program so-called Thailand healthy lifestyle plan aimed at reducing the morbidity and mortality of cardiovascular diseases and targeted on health program to promote physical activity and healthy dietary intake. Given the relationship between obesity and SES, it is particularly important to tackle the obesogenic environment and ensure that the programs reach all SES groups. The Thai Health promotion foundation, a nonprofit agency with ear mark budget from excise tax of tobacco, has sponsored national campaigns and messages on benefit of proper weight in addition to the regular programs of the Ministry of Public Health. The messages about obesity contribution to adverse health consequences have been publicized in multimedia including TV programs. However, effectiveness of these national programs and whether the messages reached all SES groups need further evaluation.

There are some limitations in the present study. Firstly, data of all age groups were not complete in all surveys. Secondly, data of causal factors such as changes in energy intake, physical activity, and factors related to energy imbalance to explain the determinants of increase in obesity were limited. Finally, the educational attainment only reflected on part of the SES and more complete data on income might add more information and get a better picture of the associations. The implication of this study is that more stringent intervention to curb the obesity trends in Thai population is needed. As obesity increases the risk of several chronic diseases which are leading to DALY loss in Thai population, without implementation of effective and integrated strategies, the burden caused by obesity will not be likely to decrease [9, 23]. Policy and environment must be designed and modified to promote healthier choice on diet and physical activity for all SES groups. Furthermore, strategies to increase the access to information on causes and burden of obesity among the lower SES group must be implemented. In conclusion, the present study demonstrated the increasing trends in BMI and obesity prevalence in all SES groups with a likelihood of higher rates among those with lower education and in rural residents during 1991–2009.

## Figures and Tables

**Table 1:** Age-specific prevalence and age-adjusted prevalence of overweight and obesity in Thai population aged ≥20 years, in 2009.

	% (95% CI)					
	Overweight (BMI 23–<25 kg/m^2^)		Obesity class I (BMI 25–<30 kg/m^2^)		Obesity class II (BMI ≥30 kg/m^2^)	
	Urban (*N* = 1,997)	Rural (*N* = 1,492)	Urban (*N* = 3,114)	Rural (*N* = 2,068)	Urban (*N* = 1,108)	Rural (*N* = 589)
All						
Age ≥ 20 years	18.2 (16.9, 19.5)	17.2 (16.1, 18.3)	28.5 (27.1, 30.0)	24.8 (22.4, 27.3)	12.2 (10.2, 14.1)	7.6 (6.2, 9.1)
Men						
All ≥20 years	19.0 (17.6, 20.5)	17.7 (15.9, 19.5)	28.2 (26.0, 30.4)	20.5 (17.3, 23.8)	9.8 (7.6, 11.9)	4.9 (3.1, 6.8)
20–34	14.8 (12.3, 17.2)	15.9 (11.9, 20.0)	21.5 (18.1, 24.8)	18.0 (13.4, 22.6)	14.4 (10.1, 18.8)	6.3 (2.7, 9.9)
35–59	21.4 (19.2, 23.6)	19.4 (17.5, 21.3)	34.4 (30.8, 38.1)	24.1 (19.7, 28.5)	7.7 (5.9, 9.5)	5.0 (3.1, 7.0)
≥60	19.4 (15.9, 22.9)	15.8 (14.2, 17.4)	26.4 (23.5, 29.3)	15.2 (12.7, 17.7)	6.0 (3.7, 8.3)	2.0 (1.4, 2.7)
Education level						
Less than primary	24.5 (13.6, 35.4)	20.5 (1.8, 39.3)	27.7 (16.0, 39.4)	3.0 (0, 6.8)	15.9 (5.9, 26.1)	5.1 (1.0, 13.9)
Primary	19.5 (15.7, 23.3)	18.6 (17.0, 20.2)	23.0 (18.9, 27.2)	18.3 (14.4, 22.2)	9.2 (4.6, 13.8)	3.8 (1.8, 5.8)
Secondary	19.5 (17.6, 21.5)	17.7 (12.1, 23.4)	29.0 (27.1, 30.8)	24.9 (21.3, 28.4)	10.3 (8.2, 12.9)	7.1 (4.2, 10.0)
University	19.4 (13.0, 25.7)	26.5 (18.1, 34.8)	34.8 (29.4, 40.1)	29.2 (18.5, 40.0)	9.9 (5.7, 14.0)	6.6 (3.4, 9.9)
Women						
All ≥20 years	17.5 (15.8, 19.2)	16.7 (15.5, 17.8)	28.9 (26.6, 31.1)	28.9 (25.8, 32.1)	14.3 (12.2, 16.4)	10.2 (8.7, 11.8)
20–34	14.8 (12.0, 17.5)	14.1 (11.7, 16.6)	20.8 (17.4, 24.3)	21.3 (16.6, 26.0)	12.9 (9.4, 16.4)	9.7 (7.1, 12.3)
35–59	18.9 (16.8, 21.0)	18.3 (16.2, 20.4)	31.8 (28.8, 34.9)	35.9 (31.2, 40.7)	15.8 (13.5, 18.0)	11.8 (9.4, 14.2)
≥60	16.6 (14.7, 18.5)	16.5 (15.1, 18.0)	33.0 (30.0, 36.0)	24.5 (21.0, 27.9)	12.4 (9.2, 15.5)	6.3 (4.2, 8.5)
Education level						
Less than primary	18.9 (4.5, 33.3)	17.8 (6.0, 29.6)	22.1 (14.2, 30.0)	28.8 (12.9, 44.7)	9.2 (2.9, 15.4)	5.4 (1.5, 9.4)
Primary	17.4 (15.1, 19.8)	17.0 (14.3, 19.8)	33.4 (27.6, 39.2)	32.7 (27.4, 37.9)	16.8 (11.8, 21.7)	9.5 (7.6, 11.3)
Secondary	17.8 (12.8, 22.8)	21.5 (15.2, 27.8)	27.8 (25.7, 29.9)	26.6 (20.7, 32.6)	13.3 (11.6, 15.1)	14.5 (9.2, 19.9)
University	17.6 (11.5, 23.7)	17.1 (10.7, 23.4)	25.9 (21.1, 30.7)	21.3 (13.4, 29.3)	9.2 (6.6, 11.8)	8.9 (3.4, 14.5)

**Table 2:** Adjusted odds ratio (95% CI) for overweight and obesity associated with educational levels in Thai adults, 2009.

	OR (95% CI)					
	Men			Women		
	Overweight (BMI 23–<25 kg/m^2^)	Obesity class I (BMI 25–<30 kg/m^2^)	Obesity class II (BMI ≥ 30 kg/m^2^)	Overweight (BMI 23–<25 kg/m^2^)	Obesity class I (BMI 25–<30 kg/m^2^)	Obesity class II (BMI ≥ 30 kg/m^2^)
	(*N* = 1,717)	(*N* = 2,118)	(*N* = 504)	(*N* = 1,772)	(*N* = 3,064)	(*N* = 1,193)
Age (per 10 years)	1.00 (1.00, 1.01)	1.00 (0.99, 1.01)	0.99 (0.98, 1.00)	1.00 (1.0, 1.01)	1.00 (0.99, 1.01)	0.99 (0.99, 1.00)
Area of residence						
Urban	1.4 (1.1, 1.7)	1.5 (1.2, 1.9)	1.7 (1.2, 2.4)	1.3 (1.1, 1.6)	1.2 (1.0, 1.5)	1.5 (1.2, 1.8)
Rural	1.0	1.0	1.0	1.0	1.0	1.0
Education level						
Less than primary	1.0	1.0	1.0	1.0	1.0	1.0
Primary	1.5 (0.8, 2.9)	2.9 (1.8, 4.8)	1.1 (0.4, 3.1)	1.7 (0.9, 3.1)	1.9 (1.4, 2.7)	2.4 (1.4, 3.9)
Secondary	1.6 (0.9, 2.9)	3.5 (1.9, 6.5)	1.6 (0.5, 4.6)	1.2 (0.7, 2.1)	1.0 (0.6, 1.6)	1.3 (0.7, 2.2)
University	2.2 (1.1, 4.7)	4.6 (2.2, 9.6)	1.5 (0.5, 4.9)	0.9 (0.4, 2.0)	0.7 (0.5, 1.0)	0.7 (0.4, 1.5)

Multinomial logistic regression model, including age, area of residence, geographic region, smoking status, and educational levels.

**Table 3:** Annual adjusted odds ratio (95% CI) for overweight, obesity class I and class II associated with educational levels in Thai adults aged 20–59 years, Thai NHES 1991–2009.

	OR (95% CI)					
	Overweight (BMI 23–<25 kg/m^2^)		Obesity class I (BMI 25–<30 kg/m^2^)		Obesity class II (BMI ≥ 30 kg/m^2^)	
	OR	*P* value	OR	*P* value	OR	*P* value
Men						
All	1.01 (0.99, 1.02)	0.07	1.04 (1.02, 1.05)	0.001	1.05 (1.01, 1.08)	<0.01
Area of residence						
Urban	1.0 (0.98, 1.01)	0.76	1.03 (1.02, 1.04)	<0.001	1.05 (1.01, 1.09)	0.02
Rural	1.01 (1.0, 1.03)	0.02	1.04 (1.01, 1.06)	0.001	1.05 (1.0, 1.1)	0.03
Education level						
Less than primary	1.01 (0.93, 1.10)	0.77	0.93 (0.88, 0.99)	0.02	1.12 (1.01, 1.25)	0.03
Primary	1.01 (1.0, 1.03)	0.01	1.04 (1.02, 1.06)	<0.001	1.03 (0.99, 1.08)	0.12
Secondary	1.0 (0.97, 1.03)	0.98	1.04 (1.01, 1.06)	0.002	1.06 (1.01, 1.12)	0.02
University	1.02 (0.97, 1.07)	0.37	1.04 (0.99, 1.08)	0.08	1.04 (0.96, 1.23)	0.33
Women						
All	1.0 (0.99, 1.01)	0.55	1.03 (1.01, 1.04)	<0.001	1.04 (1.02, 1.06)	<0.001
Area of residence						
Urban	1.01 (0.99, 1.01)	0.17	1.01 (1.0, 1.04)	0.14	1.04 (1.02, 1.06)	0.001
Rural	1.0 (0.98, 1.00)	0.16	1.03 (1.01, 1.05)	0.001	1.04 (1.02, 1.06)	0.001
Education level						
Less than primary	1.0 (0.95, 1.05)	0.55	1.03 (0.98, 1.07)	0.20	1.0 (0.94, 1.07)	0.95
Primary	0.99 (0.98, 1.01)	0.93	1.03 (1.01, 1.05)	<0.001	1.04 (1.02, 1.06)	<0.001
Secondary	1.01 (0.99, 1.04)	0.29	1.01 (0.99, 1.04)	0.25	1.05 (1.01, 1.10)	0.01
University	1.01 (0.98, 1.05)	0.36	1.02 (0.98, 1.06)	0.35	1.04 (0.98, 1.10)	0.18
